# Benign Recurrent Intrahepatic Cholestasis (BRIC): An African Case Report

**DOI:** 10.1155/2020/2894293

**Published:** 2020-03-10

**Authors:** Adil Salyani, Linda Barasa, Allan Rajula, Sayed K. Ali

**Affiliations:** Department of Medicine, Aga Khan University Hospital, Nairobi, Kenya

## Abstract

Benign recurrent intrahepatic cholestasis (BRIC) is a rare disorder characterised by recurrent episodes of cholestatic jaundice. First described in 1959, BRIC has been reported in patients all over the world including of African descent. Here, we describe a case of a 21-year-old male with recurring episodes of cholestatic jaundice where we diagnosed BRIC and terminated an episode with rifampicin. To our knowledge, this is the first case report of BRIC diagnosed in Africa.

## 1. Introduction

Benign recurrent intrahepatic cholestasis (BRIC) is a rare, autosomal recessive disorder characterised by recurrent, self-limited episodes of cholestasis manifested by pruritus, anorexia, fatigue, steatorrhea, and jaundice [1, 2]. The index episode usually occurs in the first two decades of life [2,3]. Episodes can be spontaneous or triggered by infections or pregnancy and can last from weeks to months [2,4,5]. Diagnosis is based on a compatible clinical presentation, laboratory parameters and histology with exclusion of other causes of cholestasis [2] and confirmed by genetic testing [1]. Treatment is supportive often aimed at relief of symptoms, shortening of episodes, and prevention of complications [6].

## 2. Case

A 21-year-old male of Somali descent presented to our institution in December 2018. He complained of pruritus for a month and jaundice for two weeks. The pruritus was severe, generalised, and worse at night. He also reported fatigue, loss of appetite, dyspepsia, bloating, and right upper quadrant discomfort associated with nonbilious vomiting. His urine was dark and his stools loose, fatty, and pale. There was recent easy bruising of the skin, but no mucous membrane bleeding. He had lost 6 kilograms of weight since onset of symptoms. He denied fever, arthralgia, myalgia, or rash. He had no recent drug ingestion, risk factors for acquisition of viral hepatitis, food or drug allergies, and did not smoke tobacco, consume alcohol, use injectionor inhaled drugs.

He was the second-last born of 21 siblings and half siblings and was unemployed. He reported no family history of cholestasis or liver disease. Several of his sisters were parous with gestations uncomplicated by jaundice or pruritus.

On further questioning, he reported four similar prior episodes over 7 years of varying severities lasting between two to eight weeks with intervening symptom-free periods (Table 1). These episodes were unprovoked except for two occasions triggered by cutaneous infections. The episodes followed a stereotyped pattern; the onset marked by loss of appetite and severe generalised pruritus closely followed by intense fatigue, bloating, right upper quadrant discomfort, loose fatty stools, and dark urine. Scleral icterus characteristically developed two weeks later. Termination also followed a typical pattern with sudden reduction, then resolution of pruritus, a ravenous increase in appetite with resolution of fatigue and other symptoms. Scleral icterus was the last to resolve after a delay of two weeks. These episodes were punctuated by a weight loss of 5-6 kilograms that was regained back to baseline on recovery.

On examination, we found a lean young male with scleral icterus and excoriation marks on his body. He was alert and oriented in time, place, and person. There were no stigmata of chronic liver disease. Abdominal examination revealed mild right upper quadrant tenderness with a negative Murphy's sign, a normal liver span, and no clinically detectable ascites. The rest of the examination was unremarkable.

His liver function tests revealed a cholestatic picture with markedly elevated total bilirubin, predominantly direct. Alkaline phosphatase was elevated to four times the upper limit of normal, but gamma glutamyl transferase was normal, as were the transaminases. Prothrombin time was prolonged with an INR of 1.98. Serology for hepatitis A, B, and C and HIV 1&2 was negative. These results, along with those from testing performed during the previous episodes where he did not receive a diagnosis are charted in Table 2.

An abdominal ultrasound and magnetic resonance cholangiopancreatography (MRCP) both revealed a normal liver with normal biliary and pancreatic ducts. The patient declined a liver biopsy due to financial constraints at that time. He was prescribed ursodeoxycholic acid, which he did not take. The episode resolved spontaneously after seven weeks, and follow-up liver function tests were subsequently normal.

**Figure 1:**
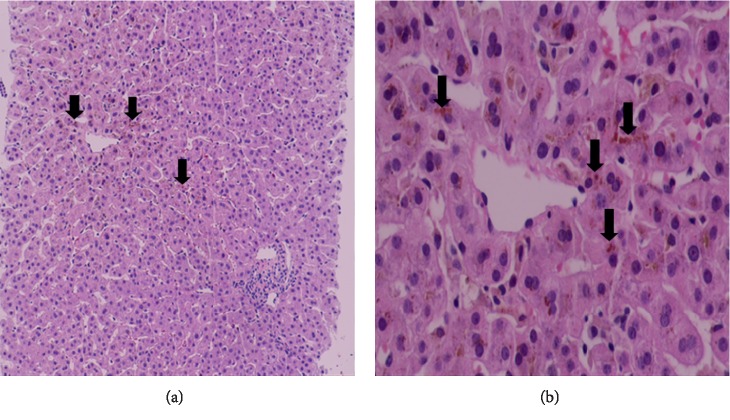
Liver histology. (a) Liver architecture is preserved. No inflammation, steatosis, or fibrosis seen (haematoxylin and eosin (H&E) × 100). Intrahepatic cholestasis predominantly in the centrilobular zone is identified (black arrows). (b) Intrahepatic cholestasis (black arrows) better demonstrated at a higher magnification (H&E* *×* *400). Central vein at the middle.

In June 2019, he presented 2 weeks into his sixth episode. Laboratory testing again revealed a cholestatic picture with peak total bilirubin 408 umol/l, direct bilirubin 326 umol/l, alkaline phosphatase 688 U/L, GGT 7 U/L, and normal transaminases. This time underwent a liver biopsy that revealed preserved liver architecture without inflammation, steatosis, or fibrosis with intrahepatic cholestasis predominantly in the centrilobular zone (Figure 1).

Based on clinical, laboratory, and histologic findings, he was diagnosed with BRIC as per the criteria proposed by Luketic and Shiffman (Table 3). He was initiated on rifampicin 150 mg twice daily for a duration of two weeks with rapid resolution of symptoms and improvement in biochemical parameters.

## 3. Discussion

BRIC, first described in 1959 in two English patients, [7] has since been reported to occur worldwide [2] including 4 patients of African descent in a 1989 case series from France and Belgium [3] and recently in a North African teenager in Switzerland [8]. While reports continue to emerge from other parts of the world [8–11], there have been no reports of cases diagnosed within Africa. This may be due to the rarity of the condition compounded by a lack of awareness of among clinicians.

Inheritance follows an autosomal recessive pattern with mutations in both alleles of ATP8B1 (BRIC1) or ABCB11 (BRIC2) [1]. Both mutations cause cholestasis by impairing the function of the bile salt export pump (BSEP), which actively transports bile into canaliculi. ATP8B1 gene codes for familial intrahepatic cholestasis type 1 transporter (FIC1), a flippase involved in translocation of phospholipids across the plasma membrane. Deficiency of FIC1 decreases plasma membrane stability, impairing the function of transmembrane transporters including the BSEP, which is encoded by ABCB11. Unlike the BSEP that is only expressed in the canalicular membrane of hepatocytes, FIC1 is also expressed in the small intestine and pancreas. This explains why extrahepatic manifestations of BRIC1 such as diarrhoea and pancreatitis are absent in BRIC2 [9].

Despite a genetic basis, most cases are sporadic. The first episode usually occurs in the first two decades of life. Episodes can last from weeks to months and can be of varying severity. In between episodes, patients remain totally devoid of symptoms for periods ranging from months to years [2, 3]. Triggers include stress, pregnancy, and airway and gastrointestinal infections [2, 4, 10, 11]. Episodes triggered by cutaneous infections seen in our patient have not been described previously.

Pruritus and jaundice are the cardinal symptoms. Other symptoms include malaise, right upper quadrant pain, anorexia, nausea, vomiting, steatorrhea, and weight loss [2]. Prolonged episodes can lead to coagulopathy and haemorrhagic tendencies due to vitamin K malabsorption [12]. Termination of the episodes is heralded by abrupt resolution in anorexia and pruritus followed by gradual resolution in jaundice. The symptoms vary from patient to patient but remain consistent in the same individual across episodes [2].

Laboratory findings during episodes reveal an elevated total bilirubin, which is predominantly direct. Alkaline phosphatase is always elevated, sometimes markedly. Transaminases are normal or mildly elevated but can also be markedly elevated [3, 13]. Early ALT rise that eventually resolves has also been described, a trend seen in our patient during his second episode [4]. The hallmark of BRIC and the other familial causes of intrahepatic cholestasis is that the GGT is normal or only mildly elevated in comparison with the other causes of intrahepatic cholestasis [2]. GGT release is associated with damage to cholangiocytes caused by elevated toxic bile salts in bile. Impaired function of the BSEP markedly reduces the concentration of bile salts in bile, thereby explaining the low levels of GGT seen in these conditions [1].

Liver biopsies performed during episodes reveal centrilobular cholestasis with bile deposition in canaliculi, hepatocytes, and Kupffer's cells. Hepatocyte degeneration, necrosis, and portal and parenchymal inflammation may be seen. These changes resolve completely between episodes [2, 10].

Diagnosis is based on the criteria proposed by Luketic and Shiffman [2] (Table 3) with genetic testing for mutations used as a confirmation [4].

Treatment is mainly supportive, aimed at relieving symptoms, shortening the episodes, and preventing complications. High dose fat-soluble vitamins should be supplemented to prevent deficiency during prolonged episodes. Bile acid sequestrants such as cholestyramine, opioid antagonists, and ursodeoxycholic acid may reduce pruritus but not the duration of episodes [2, 14]. Rifampicin, plasmapheresis, and endoscopic nasobiliary drainage have all been shown to relieve symptoms and shorten episodes [4, 15].

Rifampicin is safe and effective in reducing pruritus associated with chronic cholestasis [16]. The mechanism of action is related to its enzyme-inducing effects. It activates transcription of CYP3A4, hence stimulating 6*α*-hydroxylation of bile salts, which can be excreted at the basolateral membrane by ABCC4 transporter. It also induces UGT1A1 and ABCC2, which conjugate and excrete bilirubin [17]. In episodes of BRIC, rifampicin therapy leads to reduced symptoms, improved biochemical parameters, and termination of episodes and hence is considered first-line therapy [4, 10, 18–20].

Our case, the first reported diagnosis of BRIC in Africa, highlights the challenges faced in African countries in diagnosing such rare diseases, including lack of specialists and availability and affordability of diagnostic tests, ultimately resulting in patients receiving delayed, wrong, or no diagnosis. It also adds to the literature on safety and effectiveness of rifampicin in therapy of BRIC episodes.

## Figures and Tables

**Table 1:** Previous attacks.

Attack	Month, year	Age at onset	Trigger (s)	Duration	Severity	Period to next attack
1	2014	14	None	2 weeks	Mild	2 years
2	June, 2016	16	Skin abscesses	7 weeks	Severe	7 months
3	April, 2017	17	None	8 weeks	Severe	6 months
4	December, 2017	18	Skin abscesses	5 weeks	Moderate	11 months

**Table 2:** Laboratory results.

Investigation	Reference range	26/06/16 attack 2		09/07/16 attack 2	13/07/16 attack 2			07/01/18, 20/01/18 attack 4	04/12/18 attack 5	
White cell count (×109/L)	4–10	7.04								
Neutrophils (%)	45–75	65								
Lymphocytes (%)	25–40	26								
Monocytes (%)	2–10	7								
Eosinophils (%)	1–6	2								
Basophils (%)	0–2	0								
Red cell count (×1012/L)	4.5–6.5	5.46								
Haematocrit (%)	40–54	50.6								
Haemoglobin (g/dL)	13–18	16.4								
Platelets (×109/L)	150–400	369								
International normalised ratio (INR)									**1.98**	
Total bilirubin (umol/L)	0–21	**134.7**		**229.1**	**293.7**				**393**	
Direct bilirubin (umol/L)	0–3.4	**108.4**		**204.9**	**249.3**				**337**	
Alkaline phosphatase (U/L)	52–171	**288.5**		**313.0**	**370.0**				**692**	
Gamma glutamyl transferase (U/L)	2–42	18.1		8.0	9.0				12	
Aspartate aminotransferase (U/L)	0–50	**186.5**		**60.7**	46.1				48	
Alanine aminotransferase (U/L)	0–50	**400.4**		**62.4**	43.7				32	
Total protein (g/L)	60–85								**91**	
Albumin (g/L)	35–55								46	
Hepatitis B surface antigen	Negative	Negative							Negative	
Hepatitis A IgM antibody	Negative	Negative			Negative				Negative	
Hepatitis A total antibody	Negative				**Positive**					
Hepatitis C antibody	Negative			Negative					Negative	
Antinuclear antibody								Negative		
Antimitochondrial antibody								Negative		
Anti-LKM A^±^								Negative		
Liver antibodies^*∗*^								Negative		

^±^Antiliver kidney microsome type 1 antibody. ^*∗*^ASMA, AMA-M2, M2-3E, SP-100, PML, gp210, LKM-1, LC-1, SLA/LP, and Ro-52.

**Table 3:** Diagnostic criteria.

Diagnostic criteria for BRIC (LUKETIC and SHIFFMAN, 2004)
At least two attacks of jaundice separated by a symptom-free interval lasting several months to years
Laboratory values consistent with intrahepatic cholestasis
GGT either normal or only minimally elevated
Severe pruritus secondary to cholestasis
Liver histology demonstrating centrilobular cholestasis
Normal intra- and extrahepatic bile ducts by cholangiography
Absence of factors known to be associated with cholestasis (i.e., drugs and pregnancy)
